# A deep learning-based pipeline for large-scale echocardiography data curation and measurements

**DOI:** 10.1093/ehjdh/ztaf108

**Published:** 2025-09-17

**Authors:** Jieyu Hu, Sindre Hellum Olaisen, David Pasdeloup, Gilles Van De Vyver, Andreas Østvik, Espen Holte, Bjørnar Grenne, Håvard Dalen, Lasse Lovstakken

**Affiliations:** Circulation and Medical Imaging, Faculty of Medicine and Health Sciences, Norwegian University of Science and Technology, Postboks 8905, 7491 Trondheim, Norway; Circulation and Medical Imaging, Faculty of Medicine and Health Sciences, Norwegian University of Science and Technology, Postboks 8905, 7491 Trondheim, Norway; Circulation and Medical Imaging, Faculty of Medicine and Health Sciences, Norwegian University of Science and Technology, Postboks 8905, 7491 Trondheim, Norway; Circulation and Medical Imaging, Faculty of Medicine and Health Sciences, Norwegian University of Science and Technology, Postboks 8905, 7491 Trondheim, Norway; Circulation and Medical Imaging, Faculty of Medicine and Health Sciences, Norwegian University of Science and Technology, Postboks 8905, 7491 Trondheim, Norway; Health Research, SINTEF Medical Technology, Trondheim, Norway; Circulation and Medical Imaging, Faculty of Medicine and Health Sciences, Norwegian University of Science and Technology, Postboks 8905, 7491 Trondheim, Norway; Clinic of Cardiology, St. Olavs University Hospital, Trondheim, Norway; Circulation and Medical Imaging, Faculty of Medicine and Health Sciences, Norwegian University of Science and Technology, Postboks 8905, 7491 Trondheim, Norway; Clinic of Cardiology, St. Olavs University Hospital, Trondheim, Norway; Circulation and Medical Imaging, Faculty of Medicine and Health Sciences, Norwegian University of Science and Technology, Postboks 8905, 7491 Trondheim, Norway; Clinic of Cardiology, St. Olavs University Hospital, Trondheim, Norway; Department of Medicine, Levanger Hospital, Nord-Trøndelag Hospital Trust, Levanger, Norway; Circulation and Medical Imaging, Faculty of Medicine and Health Sciences, Norwegian University of Science and Technology, Postboks 8905, 7491 Trondheim, Norway

**Keywords:** Echocardiography, Deep Learning, DL-based measurements, Real-world Data, Datacuration

## Abstract

**Background:**

Echocardiographic image data accumulating in echo labs are a highly valuable but underutilized resource for cardiac imaging research. Despite the availability of large image databases, quantitative measurements required for clinical analysis and research remain limited. Retrospective manual measurements are highly time-consuming and susceptible to operator-related variability. Moreover, data curation and quality control metrics are needed to prepare real-world data for analysis.

**Methods:**

Deep learning-based image analysis can provide fully automated, rapid, and consistent extraction of measurements, given that the data have been properly curated. In this work, we develop an automated pipeline for data curation of a large echo database of 14 326 exams from 9678 patients and evaluate automated measurements of left ventricular ejection fraction (LVEF) and left atrial volume index (LAVI) as a use case.

**Results:**

In validation subsample of 1763 subjects with varying image quality and cardiac diseases and 1488 healthy subjects, the pipeline output was compared with manual measurements. Bland–Altman analysis revealed a bias [standard deviation (SD)] of −1.8% (7.6%) for LVEF and 3.3 mL/m² (8.1 mL/m²) for LAVI and demonstrated robust performance for varying image quality and pathological conditions. Additionally, in the large part of the database of 9678 exams without clinical measurements, the automated data curation and measurement quality control resulted in 79% measured data with high confidence.

**Conclusion:**

This work highlights the potential of deep learning-based automated measurements in echocardiography for data mining in large real-world databases, paving the way for advancements in cardiac imaging research and diagnostics.

## Graphical Abstract

**Graphical Abstract:**
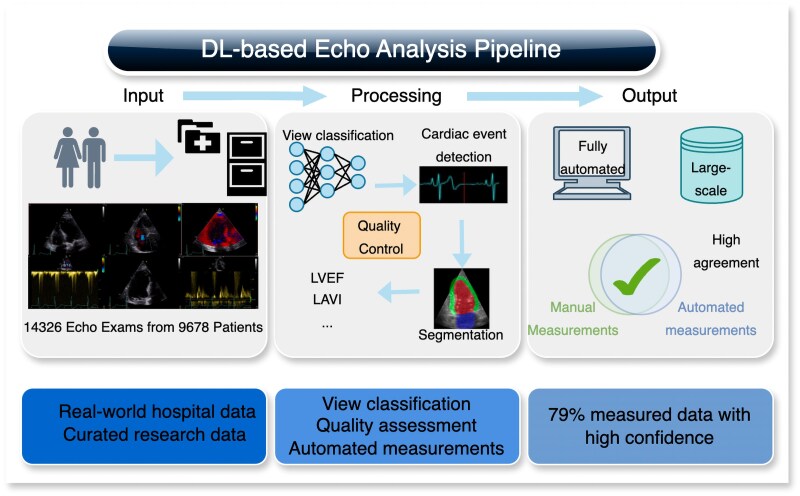
Deep learning-based echocardiographic analysis pipeline.

## Introduction

Echocardiography is widely used in clinical practice and is a cornerstone imaging modality for diagnosing patients with heart disease. Current guidelines^1–3^ recommend conducting 15 separate recordings and a high number of measurements for a standard evaluation of the left ventricle (LV) and more than 100 recordings and measurements for a complete protocol. However, practical time constraints often limit the number of quantitative measurements that can be made and visual evaluation (eyeballing) of cardiac function is common. This means that complete protocols with quantitative measurements are often unavailable. As a result, the echocardiographic data remain an untapped source of information valuable for the understanding and diagnosis of cardiac diseases. Retrospective measurements are needed to exploit the data, but this is both highly time-consuming and prone to operator-related variability. However, advancements in deep learning-based image analysis can offer methods to automatically extract quantitative measurements rapidly, robustly, and consistently and, thus, be well suited for the task of large-scale echocardiographic measurement. Previous work has demonstrated promising results in automating cardiac chamber quantification^4–7^ and for identifying myocardial dysfunction,^8,9^ as well as for assessment of prognosis.^10–13^ Two main deep learning-based approaches for evaluation of LV function include interpretable multi-step workflows including view classification, timing estimation, and segmentation of myocardial structures^14–16^ and end-to-end solutions that are less interpretable but eliminate intermediate steps.^17,18^

Analysing and searching for patterns in large-scale databases are often referred to as *data mining*. To achieve accurate and efficient mining of echocardiographic data, exams, which include a multitude of recordings, need to be filtered and prepared for analysis, a process referred to as *data curation*. Often, several recordings are used to achieve one measurement, e.g. a biplane measurement of the LV volume requires two views [apical four-chamber (A4C) and two-chamber (A2C)] with tracing of the endocardial border at the end-diastole (ED) and end-systole (ES) time points. Furthermore, several aspects challenge automated measurements in real-world clinical echocardiographic databases, as standardized views are not always available, and recordings that are not meant for measurements may also be stored. Moreover, echocardiographic image quality is influenced by factors such as operator expertise, disease characteristics, and inherent patient image quality. Thus, quality control is needed to ensure that automated measurements are done appropriately.

The main aim of this work was to investigate challenges and develop solutions for automated data curation and quality control in large echocardiographic databases. Furthermore, to validate a fully automated measurement pipeline for analysis of LV ejection fraction (LVEF) and left atrial (LA) volume index (LAVI), we present a use case in a large real-world echo laboratory dataset, where we analyse several aspects influencing measurement variability relevant for data mining.

## Overview of datasets and measurements

### Datasets

Three echocardiographic datasets were used in this work. The *TRUST* dataset consists of clinical echocardiograms extracted from the echo laboratory at the St. Olav’s University Hospital, Trondheim, Norway. Data inclusion was restricted to echocardiograms being performed by 1 of the 18 experienced personnel (sonographers, fellows, or cardiologists) in the echo laboratory during 2020–2021. In total 14326 exams from 9678 patients with mixed cardiac diseases were included. Quantitative measurements of LVEF and LAVI were available from 1763 and 982 exams, respectively. Additional information from ICD-10 diagnosis codes about cardiac diseases and comorbidities was available for all patients. More details can be found in *Table 1*.

**Table 1:** TRUST dataset description

TRUST	Female	Male	
	*n* = 3813 (39.4%)	*n* = 5865 (60.6%)	All
Age, years	66.7 ± 16.3	66.5 ± 14.1	66.6 ± 14.8
BMI, kg/m^2^	26.1 ± 5.3	26.6 ± 4.4	26.6 ± 4.6
LVEF^a^, %	48.1 ± 12.3	45.9 ± 12.5	46.0 ± 12.6
HF diagnosis, %	24.4%	24.5%	24.5%

^a^LVEF only when manual measurements were available.

Age and BMI for the whole dataset.BMI, body mass index; LVEF, left ventricular ejection fraction; HF, heart failure.

The second dataset, *HUNT4*, was gathered from a mostly healthy population in the Trøndelag county of Norway. Echo data were collected and measured by one of the two operators from 2462 volunteers. Of these, 974 were included in training of the LVEF or LAVI algorithm or had some technical issues and were excluded.^4,6^ Thus, 1488 exams from 1488 healthy individuals with available quantitative measurements of LVEF and LAVI were included.

A third already curated dataset of A2C and A4C views with the image traces used for LVEF reference measurements was just used for training the LV segmentation network. This dataset was the openly available anonymous CAMUS dataset of 500 patients.^5^ CAMUS data were not used for evaluation of the pipeline or validation, as it had limited available images and was already used for training.

All echocardiograms were acquired using GE Healthcare ultrasound scanners (Vivid E9 and Vivid E95; GE HealthCare, Horten, Norway). The regional ethics board approved all parts of the study (REC ID TRUST 7160 and HUNT 2018/2416). All patient data were de-identified in accordance with General Data Protection Regulation requirements, ensuring the removal of all personally identifiable information.

The distribution of LV volumes and LVEF for the three specified datasets is shown in Supplementary material online, *Figure S1*, for the subsets where these measurements were available. As can be observed, the clinical CAMUS and TRUST datasets have a similar LVEF distribution, while the population study HUNT4 mainly includes subjects in the normal LVEF range (>50%). Notably, though, the volumes in CAMUS and TRUST differ significantly, where CAMUS measurements are consistently lower.

### Echocardiographic examinations and measurements

Most echocardiograms from TRUST and HUNT were comprehensive and included modalities like B-mode, M-mode, continuous (CW), and pulsed-wave (PW) spectral Doppler, colour Doppler, and tissue Doppler, even though there was some variability in the TRUST database. Both 2D and 3D images were included in these two datasets, while CAMUS included just 2D B-mode images. Contrast-enhanced ultrasound images were only included in TRUST. As transthoracic echocardiography (TTE) B-mode images were the main interest for this work, we did not include images recorded using linear probes, curvilinear arrays, pencil probes, and transoesophageal cardiac probes. As several views are recorded during TTE from different acoustic windows like the apical and parasternal views with the addition of specific views zoomed in on structures of interest, there is a need to organize the TTE exams accordingly for data curation. An example of the organization of the TTE exam is shown in Supplementary material online, *Figure S2*.

Reference LVEF measurements from the TRUST and HUNT datasets were estimated from biplane measurements using two different methods, either by manual tracing of the endocardial border at ED and ES in A4C and A2C views or by semi-automated tracing (AutoEF, EchoPAC, GE Healthcare) where the software identifies the endocardial contour and tracks the endocardial border throughout the cardiac cycle. The initial contour could here be manually modified by the operator. Both methods used the Simpson method (method of discs) to estimate the volumes. For LAVI reference measurements, manual tracing and Simpson’s method were applied to A4C and A2C views at ES and then indexed to the body surface area (BSA).

## Methodology

**Figure 1:**
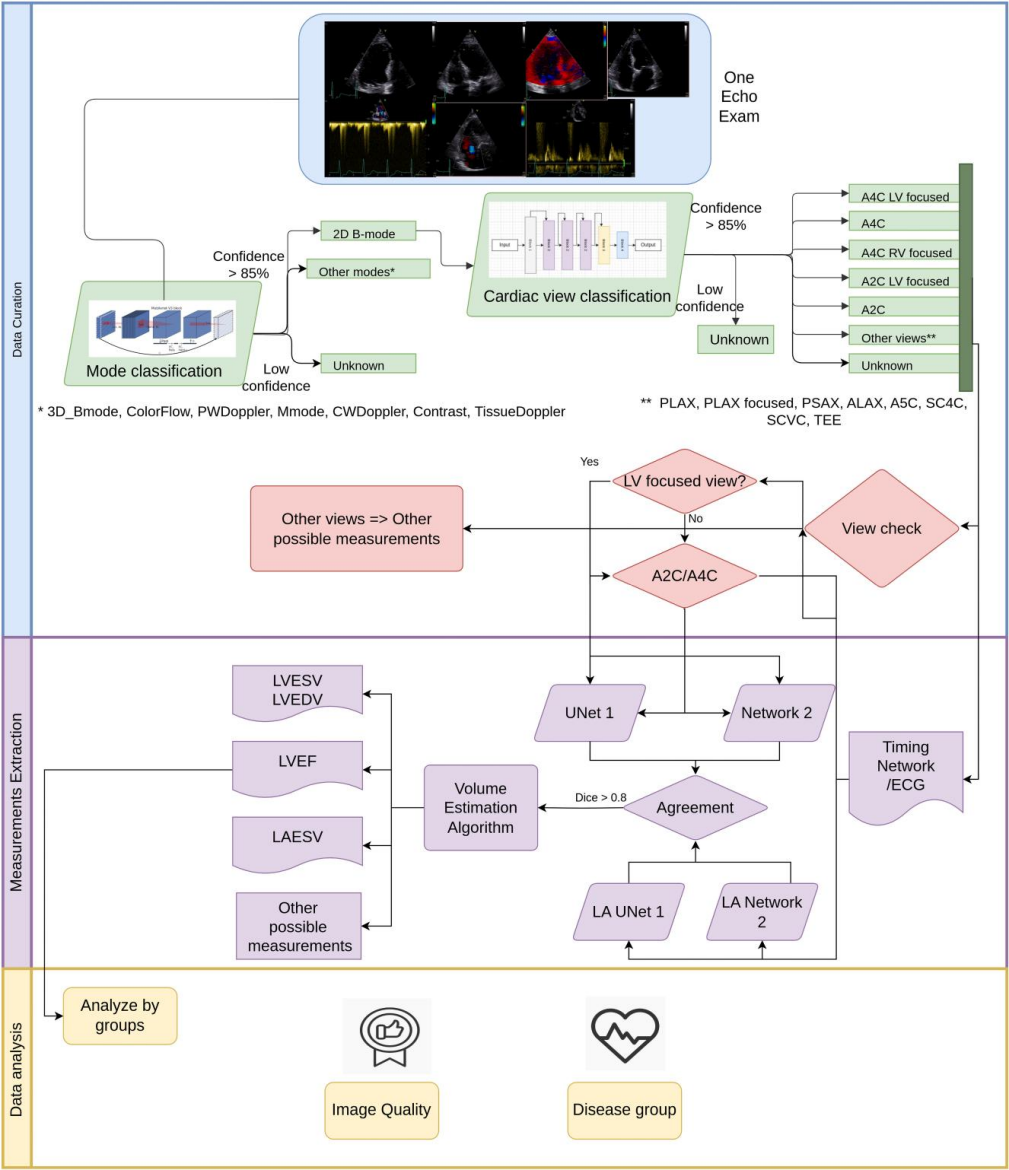
Representation of the data processing pipeline. The first stage focused on data curation including data indexing and classification. The following phase handles the measurement extraction with quality assurance mechanisms. The final part analyses the potential factors that may influence the results.

We divided the work into three phases, (i) data curation, (ii) feature extraction, and (iii) data analysis, as shown in *Figure 1* and detailed below.

### Data curation

The data curation task was divided into (i) data collection and indexing, (ii) data cleaning and classification, and (iii) data quality control.

#### Data collection and indexing

Both the TRUST and HUNT4 datasets were stored as DICOM files. Thus, the file content was stored in a structured binary format containing both metadata and imaging data. The image data and additional vendor metadata were stored in private DICOM tags (GE RawDicom), while only a single preview image was available in the public DICOM header. The DICOM metadata supports the structuring and management of large datasets in medical imaging.^19^ Raw data were accessed for research purposes through an institutional collaboration with GE HealthCare. The exams were indexed according to the DICOM Study Instance UID, unique for each exam.

#### Data cleaning and classification

The next steps were to identify the contents of the exams and extract the recordings relevant for automated measurement use cases. For these tasks, we used annotated data to train two classification networks, one for the classification of the imaging modality for each recording and another for B-mode view classification as required for fully automated measurements of LVEF and LAVI.

*Image modality classification:* A deep learning neural network was adapted from MobileNetV3^20^ and trained to categorize echocardiographic images into eight modalities; 2D B-mode, 3D B-mode, colour flow, M-mode, PW Doppler, CW Doppler, contrast-enhanced ultrasound, and tissue Doppler. The labels were acquired from private tags in DICOM files which directly provided the modality of the recordings. Preview JPEG images were resized to 224 × 224 × 3 RGB images as input. We applied the MobilNetV3Small implemented by TensorFlow with weights pretrained on ImageNet without the top layer. The last layer was modified to adapt to eight classes. The network for recognizing the image modality was trained on 17863 TRUST images (524 exams, 399 patients) and tested on 7567 TRUST images (243 exams, 201 patients). Categorical cross-entropy was used as the loss function and trained for 40 epochs.

*Detailed B-mode view classification:* To classify B-mode imaging views, we extended the CVC network^21^ to include additional view categories beyond those originally covered. The previous classification scheme included parasternal long-axis (PLAX), parasternal short-axis (PSAX), A4C, A2C, apical long-axis (ALAX), subcostal four-chamber (SC4C) and vena cava inferior (SCVC), and an unknown class. We extended the classification to have additional specialized views, PLAX focused (zoomed on the LVOT), A4C LV-focused, A2C LV-focused, apical five-chamber (A5C), and further transoesophageal echocardiographic recordings (TEE). DICOM preview images were resized to 224 × 224 grayscale images and used as input. The network was trained on 14451 TRUST images (2059 exams, 1886 patients) and tested on 6152 TRUST images (489 exams, 482 patients).

*Image quality classification:* Image quality was estimated using the open-source arqee software recently developed by our group, based on a deep learning model trained using expert annotations of regional image quality in apical views of 311 HUNT4Echo participants.^22^ The network provides a metric of image quality per segment for apical views, which we averaged to provide a global image quality of the A4C and A2C views. The quality scores were rounded to a value from 0 to 5, with 0 representing the lowest quality and barely visible cardiac structures and 5 indicating the highest quality with clearly identified cardiac structures as the endocardial border, the myocardium, and the valves.

#### Data quality control of classification of image modality and B-mode views

Quality control of the output from the modality and view classification networks was done by thresholding the confidence value at the output classification layer. For each view classification, a predefined confidence value higher than 85% was required to validate data for further analysis and measurement.

### Measurement extraction

Based on the automated exam organization and the overview of present modalities and 2D views, we generated a list of possible image measurements to be extracted based on established guidelines.^1–3^ For this paper the measurements were limited to the biplane measurements of LV and LA volumes and LVEF. Only A4C and A2C were used, and LV measurements were performed at ED and ES with calculation of volumes and LVEF, while LAVI measurements were done at ES. Both the timing and segmentation were obtained by separate deep learning model networks as described below, and subsequently measurements were calculated based on Simpson’s method. When several recordings of the same view were present, or when multiple cardiac cycles were available for a given recording, the average of several measurements was provided.

#### Timing of cardiac events and measurements

End-diastole and ES were defined as the closure of the mitral and aortic valves, respectively. These time points were automatically extracted by a previously developed cardiac event detection model trained on echocardiographic B-mode data.^23^ In cases where the cardiac event detection model failed to identify ES and ED, we relied on the digital 3-lead ECG present in the DICOM file, where the timing was defined based on the following known regression formulas^24^:


$$\begin{array}{c} \mathrm{ES}=\mathrm{ED}+498-1.6\times \mathrm{HR}\;[\mathrm{Male}]\\ \mathrm{ES}=\mathrm{ED}+522-1.77\times \mathrm{HR}\;[\mathrm{Female}] \end{array}$$

where ED and ES represent the timing in milliseconds and HR is the heart rate in beats per minute derived from the ECG trace.

#### Segmentation and volume calculation

The segmentation model of the LV and LA was a U-Net developed and validated in our previous work^4,6^ but here extended by also training it on the CAMUS dataset^5^ to include more data variability. A second model, GraphNet, was used for quality control, below and in.^25^ The LA segmentation network was developed and validated in previous work.^6^ Biplane volume estimates were calculated based on the segmentation of the LV or LA using the Method of Disks (Simpson’s method).

LVEF is defined as follows:


$$\mathrm{EF}=\frac{\mathrm{EDV}-\mathrm{ESV}}{\mathrm{EDV}}\cdot 100\text{\%}$$

where EDV is end-diastolic LV volume and ESV is end-systolic LV volume.

LAVI is defined as follows:


$$\mathrm{LAVI}=\frac{\mathrm{LAESV}}{\mathrm{BSA}}$$

where LAESV represents the biplane volume of the left atrium measured at ES and BSA is the body surface area as follows:


$$\mathrm{BSA}=\frac{\sqrt{\mathrm{Weight}(\mathrm{kg})\times \mathrm{Height}(\mathrm{cm})}}{60}m^{2}$$

#### Quality control mechanisms for the measurement-related procedures

Only clips with a view classification confidence score ≥0.85 were included in the analyses. If more clips from the same view exceeded the predefined cut-off the measurements from all available clips were averaged.

The timing model was quality controlled by comparing it with the output of the ECG-based regression formula. If the timing network failed to provide a valid output at all, or if the time points were more than the predefined cut-off of five frames away from the output of the regression formula, we relied on the latter for determining the required time points for EF calculations.

For the quality control of the segmentation networks, we used a dual network approach as described previously,^25^ where the agreement of two different segmentation network topologies (U-Net and GraphNet) serves as a quality metric, since they have different characteristics when faced with challenging or out-of-distribution data. During the validation process, we calculated the Dice score between the segmentation masks from the two networks. We used a predefined arbitrary Dice score ≥0.8 for inclusion of data, as we considered this a strong indicator of robust segmentation.

### Data validation and analysis

#### Validation of the data curation pipeline

The data curation pipeline was tested using echocardiographic exams from the TRUST dataset that included 1763 exams (1560 patients) with manual LVEF measurements and 982 exams with LAVI measurements. For LVEF measurements, a hierarchical approach based on the classification results was implemented. Left ventricular-focused views were primarily utilized for LVEF measurements due to their clear visualization of ventricular structure and reduced possibility of LV foreshortening, which could otherwise underestimate the true volume. When focused views were unavailable, standard views served as an alternative for LVEF calculations. For LAVI measurements, standard views that provide visualization of the left atrium were used.

#### Validation of the measurement pipeline

Validation of the automated measurement pipeline was performed by comparing the output with reference measurements from clinical experts. For LVEF and LAVI validation, we analysed both the subset of the TRUST dataset (representing real-world hospital echo data, 1763 exams) and 1488 exams from healthy individuals in the HUNT4 dataset.

The agreement between automated and reference measurements was assessed using Bland–Altman analysis. For each dataset, we calculated bias (mean difference between automated and reference measurements) and limits of agreement (LOA; defined as ±1.96 standard deviations from the mean difference). These metrics were calculated separately for each dataset and then compared to evaluate the pipeline's consistency across diverse patient populations.

#### Analysis of a large real-world echo database

To gain insight into the performance of the automated data curation and measurements, we first analyse and exemplify cases where our measurement pipeline deviates substantially from the manual reference measurements. This deviation could be due to the failure of one or more pipeline components or due to invalid reference values. We further analyse the pipeline performance for varying image quality, using the image quality network described above to separate the data into different image quality categories and report results for each. We finally analysed the performance of the measurement pipeline to patient disease profiles, where we grouped data according to the main (ICD-10) diagnosis codes. Patients were here grouped based on having ischaemic heart disease, arrhythmia, heart failure (HF), and valvular disease.

## Results

### Data curation and validation

#### Validation of data curation tools

**Figure 2:**
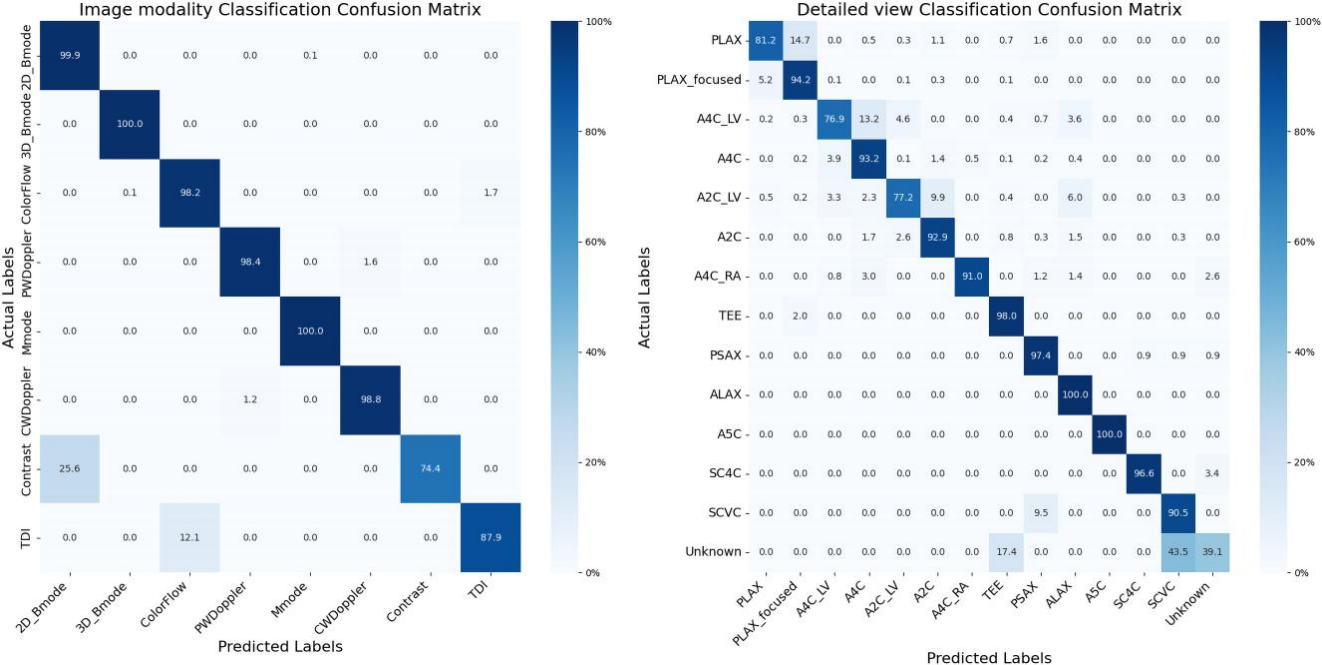
Confusion matrix of the two classification networks. The left one is for image modality classification and the right one is for detailed B-mode view classification.

In the test set of 7567 TRUST images, the classification network for image modality achieved a weighted accuracy of 98%, demonstrating robust performance (*Figure 2*). The main misclassification was contrast-enhanced images classified as 2D B-mode (*n* = 10, 25.6%) and tissue Doppler classified as colour Doppler images (*n* = 22, 12.1%).

The classification network used to identify the main B-mode view reached 90% weighted accuracy. False positives were mainly observed for the ‘unknown’ class, and some classification errors were also seen for focused and unfocused LV related to variations of image depth.

**Figure 3:**
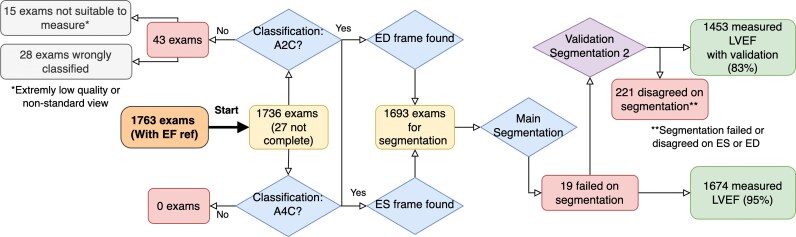
Flowchart depicting the feasibility results of the subset of our dataset with manual left ventricular ejection fraction reference measurements. The final percentage represents the feasibility.

A flow chart depicting the data curation, quality control, and validation is shown in *Figure 3*. The automated data curation pipeline analysed 1763 TRUST exams where manual measurements existed. From these, 27 exams were correctly excluded due to missing B-mode recordings in the dataset, while 43 seemingly lacked A2C views required for further analysis. By manual inspection, we found that 15 were correctly excluded but that 28 exams were wrongly excluded due to classification errors. The timing network missed 8.4% of the recordings for ES and 17.3% for ED. We replaced missing or deviating output from the timing network with ECG-based timing information (regression formula), and no exams were lost in this process. In the final step, 19 exams failed to output a valid segmentation, and 1674 exams (95%) could initially be used for analysing the measurement pipeline. When adding a second segmentation network as a quality control step, this was reduced to 1453 exams (82%), further detailed below. The exams remaining after data curation included both A4C and A2C views for LV volumes and LVEF calculations. Furthermore, based on the same dataset, 982 exams included suitable A4C and A2C views for measuring the LA volume.

#### Measurement pipeline validation

##### Cardiac event detection

We evaluated the cardiac event detection model by comparing it to the more conservative regression formulas based on the ECG trigger information available in the 1693 TRUST exams defined above. The differences between the network output and the regression formula were on average 50.5 ± 54.9 ms, corresponding to 2.97 ± 3.27 frames. Separately, values were 40.7 ± 49.9 ms (2.37 ± 3.03 frames) for ES and 58.1 ± 57.3 ms (3.43 ± 3.37) for ED. In the TRUST validation dataset, the feasibility of the timing network was 92% for ES and 83% for ED. In the analysis, we resorted to the ECG and regression formula in the cases where the timing network failed.

##### LVEF and LAVI measurements

**Figure 4:**
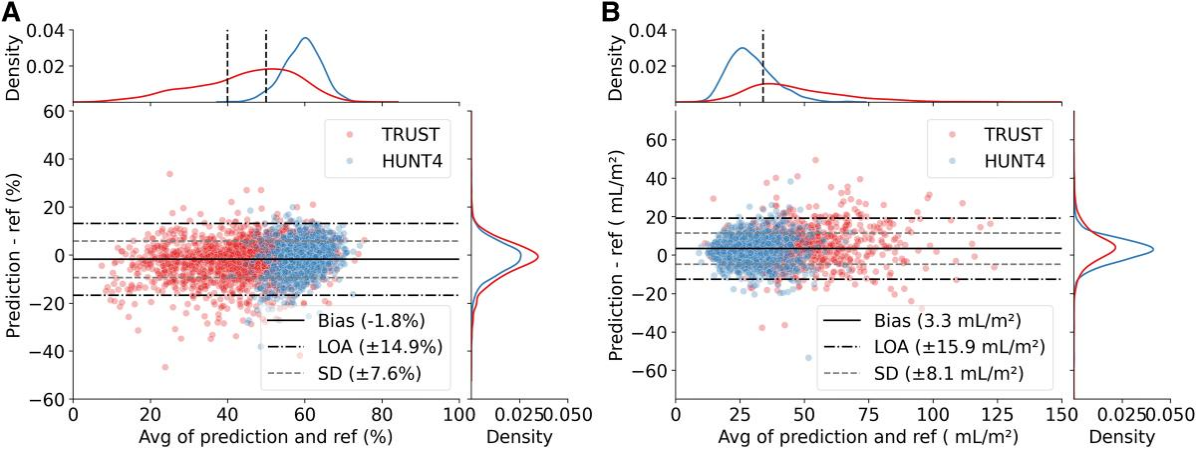
Bland–Altman plots with limits of agreement [LOA; bias ± 1.96 standard deviations (SD)] between reference and our pipeline’s estimation of (*A*) left ventricular ejection fraction and (*B*) left atrial volume index from TRUST and HUNT4 datasets, respectively. The distribution of observations is shown on top and to the right by kernel density estimate plots. Black lines represent bias, SD and LOA of our pipeline compared with the reference derived from the combined datasets.

The agreement between the measurement pipeline’s output and reference measurements is shown in *Figure 4*. For LVEF measurements, the mean differences (SD) were −2.0% (8.2%) in TRUST and −1.4% (6.7%) in HUNT, respectively. The overall bias was −1.8% (7.6%) with LOA of ±14.9%. Corresponding values for LAVI were 4.6 mL/m² (9.9 mL/m²) in TRUST and 2.5 mL/m² (6.5 mL/m²) in HUNT, respectively. The overall bias was 3.3 mL/m² (8.1 mL/m²) with a LOA of ±15.9 mL/m².

##### Feasibility and outliers

After the segmentation quality control using a second LV segmentation network (GraphNet), 221 exams were excluded. In the resulting 1453 exams, the mean (SD) difference for LVEF was slightly lower, −1.6% (7.3%), suggesting a trade-off between feasibility and agreement. Supplementary material online, *Figure S3*, illustrates some common sources of errors, including incorrect view classification for volume calculations, segmentation errors, and the selection of incorrect frames as ES or ED.

### Variability analysis

#### Image quality analysis

**Figure 5:**
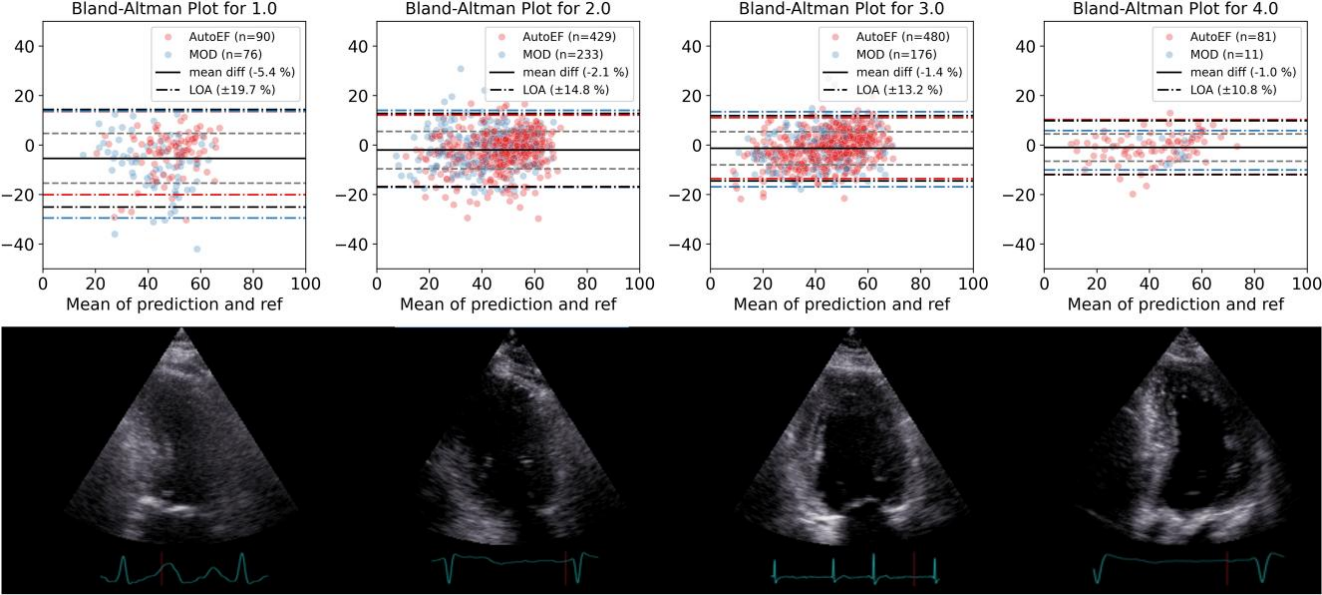
Bland–Altman plots according to categories of lowest to highest image quality. Limits of agreement are shown for reference measurements by the manual method of disc summation (MOD) and the semi-automated method (AutoEF). The black dashed line represents the standard deviation of both reference methods. Abbreviations: AutoEF, semi-automated method; MOD, method of disc summation (manual reference); diff, difference; LOA, limit of agreement.

*Figure 5* displays Bland–Altman plots grouped by image quality score classes, from 1 (lowest image quality) to 4 (highest quality). Class 0 and 5 are not included as only two cases belonged to class 0 and no cases for class 5. We observed a trend towards reduced measurement variability from low to high image quality. For LVEF the LOA was ±19.7 in the group with the worst image quality and ±10.8% in the group with the best image quality. When image quality was poor, the semi-automated method (AutoEF) showed slightly better agreement with the pipeline measurements compared with when fully manual measurements were used as reference, while the opposite was found for the best image quality group.

#### Agreement across disease groups

In *Table 2*, the bias and standard deviation for LVEF and LAVI measurements are given for different disease groups. Note that disease groups partially overlap as these diseases usually interact and co-exist. The differences between the disease groups are small, indicating that the automated pipeline is robust and not affected by specific patient conditions.

**Table 2:** Bias and standard deviations between our pipeline’s estimated left ventricular ejection fraction and left atrial volume index references for different patient groups. (*N* = 1285 for left ventricular ejection fraction, *N* = 728 for left atrial volume index)

Bias and Std (LVEF)			Bias and Std (LAVI)		
Diseases	With the condition	Without the condition	Diseases	With the condition	Without the condition
Ischaemic (*n* = 802, 483)	−1.7% ± 7.6%	−1.7% ± 8.2%	Ischaemic (*n* = 432, 296)	4.4 ± 8.7 mL/m2	4.1 ± 10.7 mL/m2
Arrhythmia (*n* = 673, 612)	−2.3% ± 8.2%	−1.1% ± 7.3%	Arrhythmia (*n* = 397, 331)	4.6 ± 10.9 mL/m2	3.9 ± 7.7 mL/m2
Heart failure (*n* = 632, 653)	−2.1% ± 7.6%	−1.4% ± 8.0%	Heart failure (*n* = 352, 376)	4.7 ± 10.5 mL/m2	3.9 ± 8.6 mL/m2
valve disease (*n* = 414, 871)	−2.1% ± 8.9%	−1.6% ± 7.2%	Valve disease (*n* = 238, 490)	5.3 ± 10.8 mL/m2	3.8 ± 8.9 mL/m2
None of these diseases (*n* = 69)	−1.5% ± 6.2%		None of these diseases (*n* = 69)	2.6 ± 5.9 mL/m2	

LVEF, left ventricular ejection fraction; LAVI, left atrial volume index.

**Figure 6:**
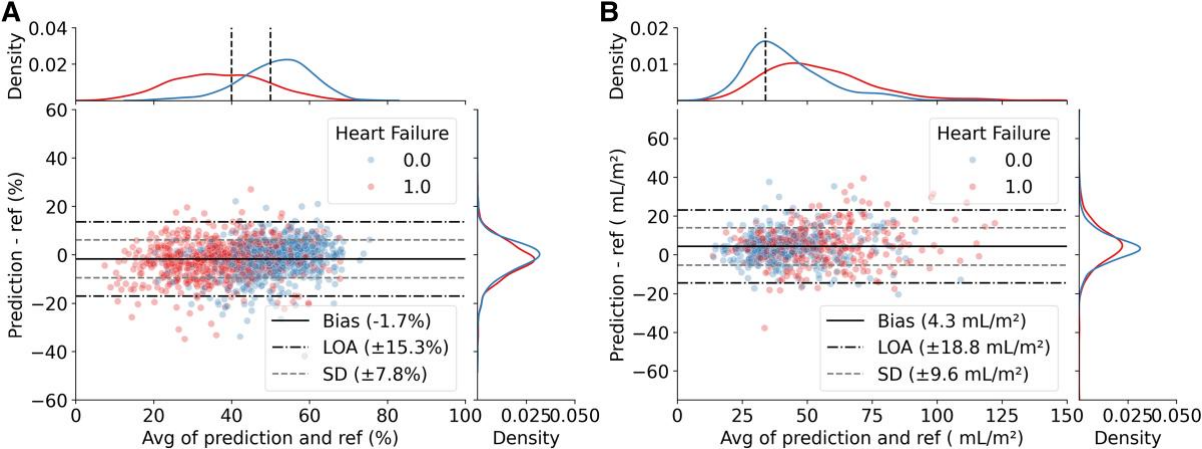
Bland–Altman plots with kernel density estimate plots between reference and pipeline estimations of *A*) left ventricular ejection fraction, *B*) left atrial volume index for subgroups with (1.0) and without heart failure (0.0). Explanations and abbreviations as in *Figure 4*.

It is important that the automated measurements are consistent across the range of values met in the clinic. We here specifically analyse this aspect for HF patients, where, for instance, specific cut-offs in LVEF are used to guide treatment. In *Figure 6*, we compare a cohort of patients (*n* = 1285 for LVEF and 728 for LAVI) with and without HF. The distribution of LVEF and LAVI is different, however, with similar LOA across the data range.

### Measurements in the large unlabelled dataset

We finally applied the data curation and measurement pipeline to measure the full 14326 TRUST exams without manual measurements. The data curation pipeline excluded 707 transoesophageal echocardiography exams, resulting in 13619 exams for further analysis. The proposed pipeline with a GraphNet as segmentation quality control was utilized and resulted in 8283 LVEF measurements and 8868 LAVI measurements. Supplementary material online, *Figure S4*, shows the distribution of the predicted LVEF and LAVI compared with the small fraction of exams with reference measurements. The distributions were similar, indicating that the automated pipeline produces consistent results at the average level.

## Discussion

In this study, we investigated opportunities and challenges for automated data curation and measurements in a real-world echocardiography database. We developed DL-based tools for organizing clinical echocardiographic exams consisting of many recordings from different ultrasound modalities and views and suggested quality control mechanisms to exclude the data which were not suitable for measurements. The automated measurement pipeline for LVEF and LAVI was based on previous published work in our group,^4,6^ which we here extended with a new validation study for real-world data.

The classification networks for organizing exam recordings proved to be robust for our purpose of extracting measurements from the B-mode recordings, with accuracy >90%. More training data may be needed for future purposes, such as the use of Doppler images. We trained the networks using DICOM preview images, as they are readily available for all vendors. Although it can theoretically be applied beyond GE HealthCare systems, we acknowledge a limitation of this study that we have not evaluated the performance on other vendors. Future validation across multiple vendor platforms would strengthen the generalizability of our approach. Improved results may be obtained by including more frames per cardiac cycle as done in previous work.^21^ In the TRUST validation dataset of 1763 exams, the data curation steps removed 5 or 15%, depending on the use of the segmentation quality control step previously proposed.^25^ By manual inspection we found that the main disagreement between segmentation networks was for data with low image quality. While quality control is essential for reliable measurements, excluding lower quality data may reduce dataset representativeness.

A positive association between higher image quality and measurement accuracy was observed, with LVEF measurement variability nearly doubling from the highest to lowest quality groups. This finding aligns with our previous works that outliers where automated methods failed commonly have low image quality.^4,6^ The image quality classification could thus also be used as a quality control component to ensure accurate results. However, this should be done with care, as image quality is also linked to patient characteristics (e.g. BMI) and could result in biased data selection. It is further expected that the reference values are more inaccurate for low image quality, and it remains to be shown if automated measurements can help in this scenario.

In our comparison, we found that the timing network predictions were approximately three frames different than the ECG-based regression approach on average, where the frame rate generally was >50 Hz. We here resorted to both methods to get ED and ES timing for the measurements in our dataset, resulting in better feasibility but potentially lower accuracy. We assume that LVEF and LAVI measurements are not highly sensitive to timing precision, though this requires further validation. For other measurements this is not the case, such as for strain-based measurements involving more accurate timing of valve events. The reason that the pipeline failed and resorted to the regression formula mostly for ED is related to the presence of only one cardiac cycle providing only a few frames prior to the ED event.

The automated measurement pipeline for LVEF and LAVI was here applied in a real-world echo database. This differs from clinical study data, which is typically more standardized and acquired with less time constraints. Comparing our pipeline’s results in the TRUST and HUNT4 databases, we observed similar performance, indicating a high robustness after data curation. The main differences were observed for LAVI, which partly can be attributed to the more standardized LA-focused recordings used in the HUNT4 dataset. The results for LVEF in HUNT4 were slightly worse than what we found previously,^4^ which can be attributed to the fully automated data selection and curation done for this study.

The robustness of the approach was further investigated by evaluating the measurement performance for various patient disease profiles. We observed little to no difference in bias and LOA between patients with ischaemic, arrhythmia, valve disease, and HF diagnoses. Furthermore, when inspecting the range of measured LVEF for HF patients in *Figure 6*, we observe consistent measurements over the range of EF values, with little or no heteroscedasticity. It should be noted that the patient disease groups here partially overlap, as multiple conditions often co-exist.

We measured the full TRUST database from 2020 and 2021. Here we have no reference measurements, but as can be observed in Supplementary material online, *Figure S4*, the distributions in both LVED and LAVI are similar to those with measurements. However, this agreement does not guarantee accuracy for individual measurements, which may still contain substantial variability and should be interpreted with appropriate clinical context. Considering that a manual biplane EF analysis can take more than 5 min,^4^ measuring 10 000 exams will take at least 800 h. Adding to this operator intra-/inter-observer variability, the automated deep learning-based approach has clear advantages. While we have not investigated if the automated pipeline provides more consistent and reproducible measurements, we have shown that this indeed is the case for both LVEF and strain measurements in our previous work.^4,8^

In summary, our automated data curation and measurement pipeline provides robust and accurate LVEF and LAVI measurements. These were chosen as representative use cases for this study. Importantly, this work can easily be extended to include complete echocardiographic analyses in the future. The pipeline can be exploited to extract measurements from echo data on a large-scale for the purposes of clinical research and for data mining large echo databases. Our future work will aim to investigate the mapping between echo markers and patient diagnosis and outcome, to learn more about cardiac disease and to better exploit echocardiography for diagnosis, risk assessment, and treatment planning of individual patients in the future.

## Conclusion

Our fully automated deep learning-based pipeline for data curation and automated measurements can provide echocardiographic measurements of LVEF and LAVI from large echo databases. The robust performance across image quality and pathological conditions highlights its adaptability for real-world usage. By automating measurement extraction, these tools may contribute to the standardization of measurements and provide a more detailed and large-scale analysis of the complex relationship between imaging phenotypes and cardiac disease. In the long run, this advancement could make echocardiography an even more impactful tool for cardiovascular research and patient diagnosis.

## Supplementary Material

ztaf108_Supplementary_Data

## Data Availability

The TRUST and HUNT4 datasets used in this study are not publicly available due to privacy and ethical restrictions. The CAMUS dataset^5^ is publicly available at https://www.creatis.insa-lyon.fr/Challenge/camus/databases.html.
