# Supplementary material for: A deep learning-based pipeline for large-scale echocardiography data curation and measurements
Source: Eur Heart J Digit Health. 2025 Sep 17;6(6):1194–203. doi: 10.1093/ehjdh/ztaf108 (PMC12629651; doi:10.1093/ehjdh/ztaf108)
Supplement: ztaf108_Supplementary_Data [file ztaf108_supplementary_data.docx]

Supplementary material:


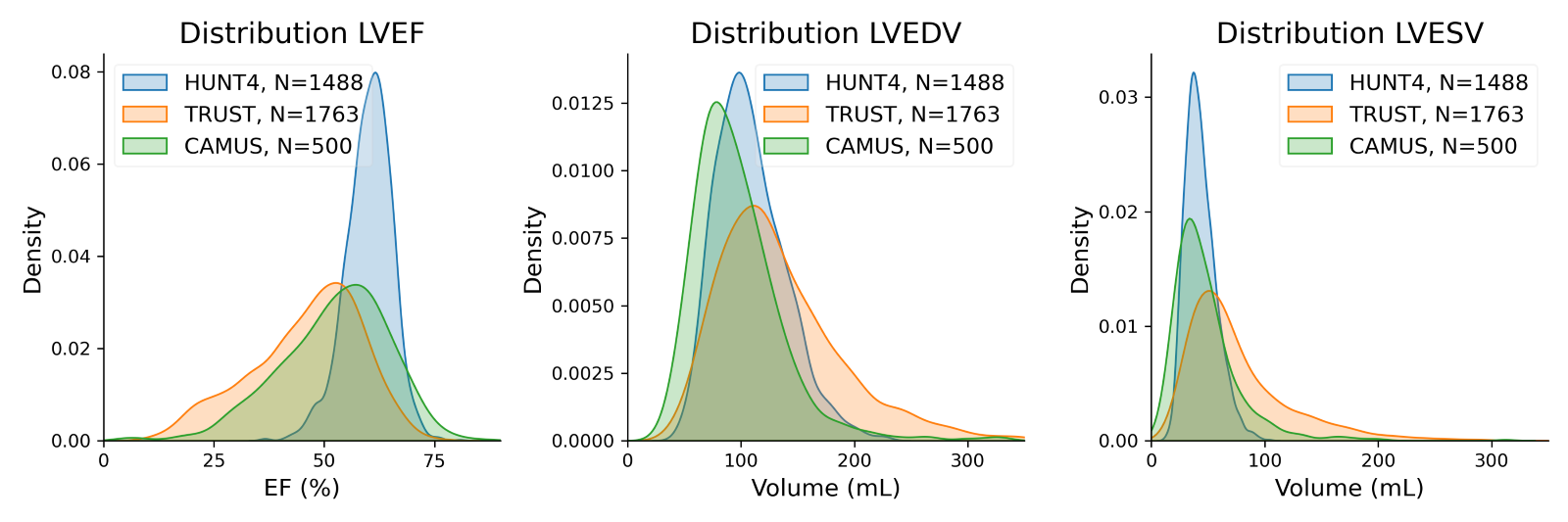


**Figure 1.** Distribution of the left ventricular ejection fraction and volumes in different datasets


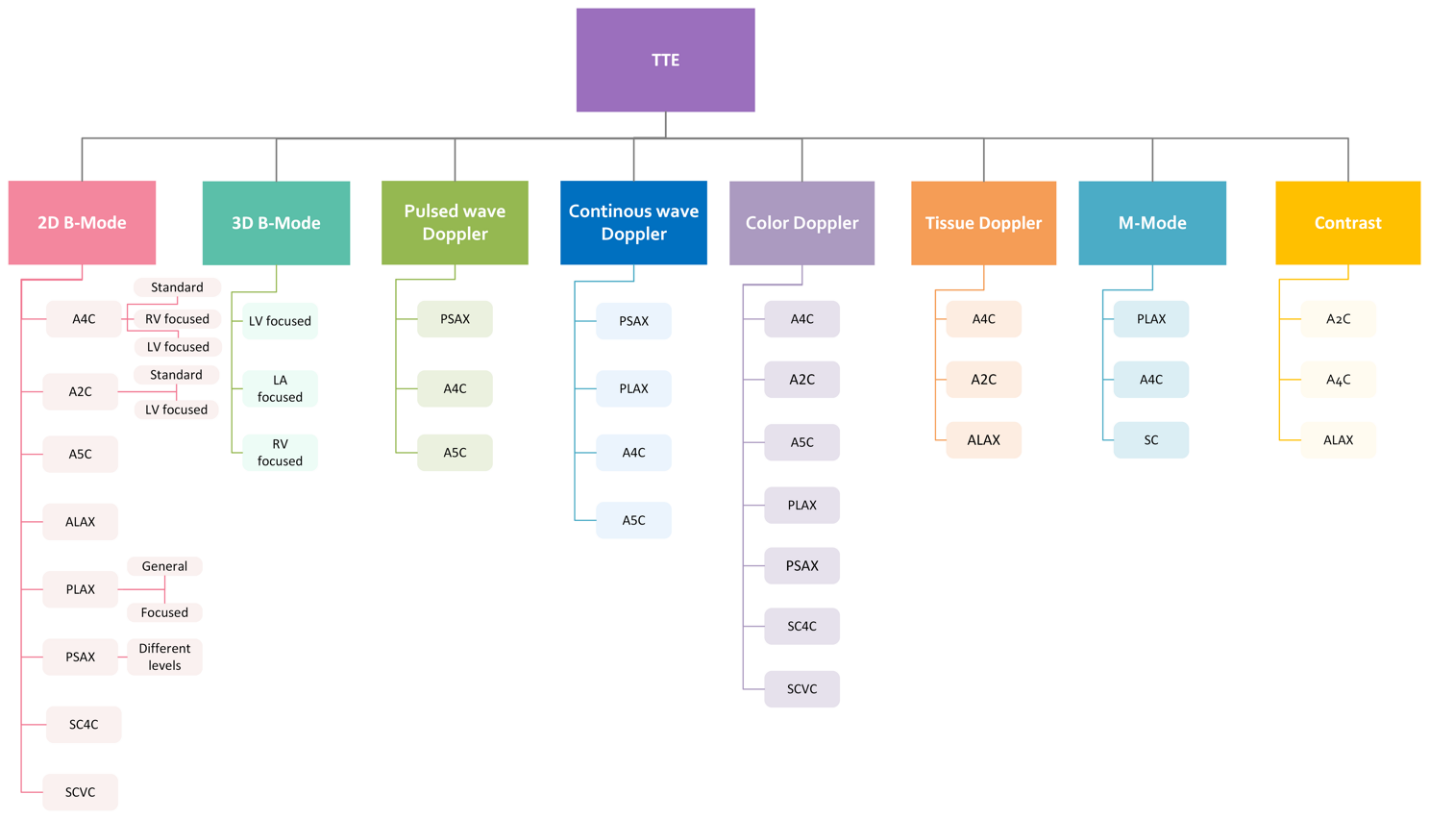


**Figure 2.** Representative example of transthoracic echocardiography (TTE) with 2D, 3D, M-Mode, Doppler imaging and contrast enhanced 2D imaging to assess heart structure and function. Key views include Parasternal Long-Axis (PLAX), Parasternal Short-Axis (PSAX), Apical Four-Chamber (A4C), Apical Two-Chamber (A2C), Apical Long-Axis (ALAX), Apical Five-Chamber (A5C), Subcostal Four-Chamber (SC4C), Subcostal Inferior Vena Cava (SCIVC), and Subcostal views. These views provide detailed images of heart chambers including Left Ventricle (LV), Right Ventricle (RV), Left Atrium (LA), Right Atrium (RA), and valves, as well as blood flow.


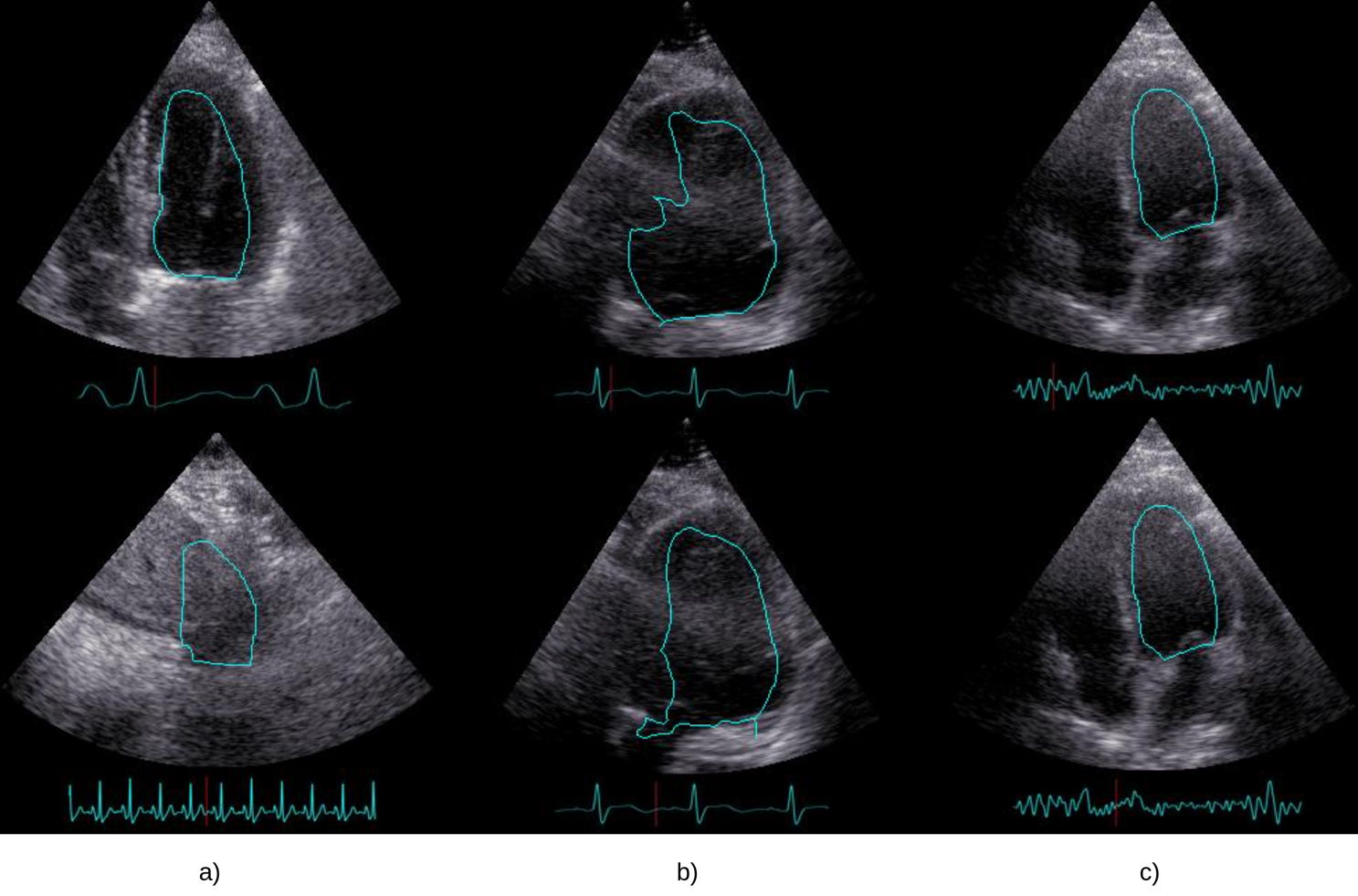


**Figure 3.** Examples of outliers in the validation dataset, and reasons for the errors. a) The lower panel image is wrongly classified causing a biplane volume calculation error, b) erroneous segmentation due to poor image quality, c) arrhythmia case where the cardiac event detection was less precise.


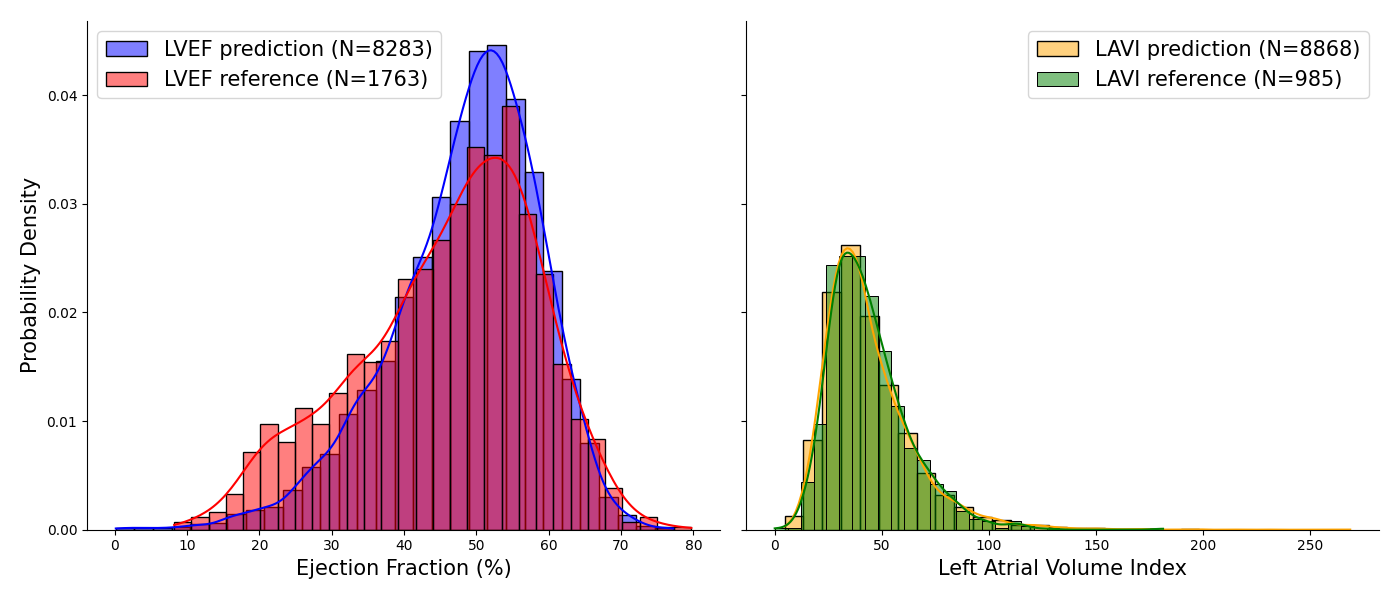


**Figure 4.** Distribution of LVEF and LAVI values by the automated pipeline and reference measurements. For the predicted LVEF measurements GraphNet was used as second validation network to ensure the best accuracy.
